# GenomeD3Plot: a library for rich, interactive visualizations of genomic data in web applications

**DOI:** 10.1093/bioinformatics/btv376

**Published:** 2015-06-20

**Authors:** Matthew R. Laird, Morgan G.I. Langille, Fiona S.L. Brinkman

**Affiliations:** ^1^Department of Molecular Biology and Biochemistry, Simon Fraser University, Burnaby, British Columbia, V5A 1S6 and; ^2^Department of Pharmacology, Dalhousie University, Halifax, Nova Scotia, B3H 4R2, Canada

## Abstract

**Motivation:** A simple static image of genomes and associated metadata is very limiting, as researchers expect rich, interactive tools similar to the web applications found in the post-Web 2.0 world. GenomeD3Plot is a light weight visualization library written in javascript using the D3 library. GenomeD3Plot provides a rich API to allow the rapid visualization of complex genomic data using a convenient standards based JSON configuration file. When integrated into existing web services GenomeD3Plot allows researchers to interact with data, dynamically alter the view, or even resize or reposition the visualization in their browser window. In addition GenomeD3Plot has built in functionality to export any resulting genome visualization in PNG or SVG format for easy inclusion in manuscripts or presentations.

**Results:** GenomeD3Plot is being utilized in the recently released Islandviewer 3 (www.pathogenomics.sfu.ca/islandviewer/) to visualize predicted genomic islands with other genome annotation data. However, its features enable it to be more widely applicable for dynamic visualization of genomic data in general.

**Availability and implementation:** GenomeD3Plot is licensed under the GNU-GPL v3 at https://github.com/brinkmanlab/GenomeD3Plot/.

**Contact:** brinkman@sfu.ca

## 1 Introduction

In the study of an organism’s genome multiple data sources are often integrated along side the genome to facilitate analysis (Langille *et al.*, 2009). Two often used methods for visualization are linear or circular representations of the genome stacked with this additional data as seen in tools such as GMOD (Stein *et al*, 2002) or Circos (Krzywinski *et al.*, 2009). However these often create either static image or visualizations with limited interaction not taking advantage of the advances in web browsers and their associated javascript engines. Newer tools have been produced such as JBrowse (Skinner *et al.*, 2009) that bring dynamic interaction to these datasets, but unfortunately act more like a standalone tool, being complex to setup and not integrating seamlessly in to existing analysis platforms. Other tools such as Scribl (Miller *et al.*, 2013) and Genome Maps (Medina *et al.*, 2013) lack a circular genome view, which is useful for some types of microbial analyses (Dhillon *et al.*, 2013).

GenomeD3Plot takes advantage of the well supported D3 library (http://d3js.org/) used by hundreds of websites globally to allow rapid creation of dynamic, interactive visualizations of datasets. The goal in the development of GenomeD3Plot was to create a library with minimal external dependencies that could be integrated in to existing web applications just as a developer might include an image or table. The use of JSON, a standardized and well supported data format, to configure a visualization with GenomeD3Plot reduces the burden on developers and lowers the complexity of use.

## 2 Features

GenomeD3Plot features an easy to use JSON based data language for visualizations. In its simplest form, a developer needs only define the tracks, give the size of the genome and the base pairs boundaries of elements to visualize, GenomeD3Plot will create an SVG canvas and plot the genomes and associated metadata as either a linear or circular view 

### 2.1 Track types

GenomeD3Plot contains four track types that may be displayed:

*Standard Track*—a set of start and end base pair to visualize regions of a genome
*Stranded Track*—similar to a standard track but with an additional strand parameter for each region to visualize the region above or below the mid-point of the track
*Plot track*—A series of base pairs of equal distance to visualize continuous data such as average GC content
*Glyph Track*—Features to be shown at a specific base pair, with the ability to dynamically stack as needed in a crowded visualization.

### 2.2 API

GenomeD3Plot features a rich API to allow developers to dynamically manipulate visualizations. Tracks can be hidden and added back, the radius of tracks in a circular plot can be shifted, or the plot can be zoomed or resized. To assist in seeing visualization updates, elements animate in and out or to new locations on the plot.

In addition, callbacks can be configured to call javascript functions or objects on mouseover, mouseout and click events allowing for a rich interaction experience with other existing website elements.

With just two function calls, a linear and circular plot can also be tied together so that manipulation of one will cause a mirror alteration in the other, such as zooming or changing the visible region of the genome.

### 2.3 Interactivity

GenomeD3Plot responds to drag and mousewheel events to control the zoom level and visible region of a linear genome plot. In addition there are optional drag bars to allow users to dynamically resize the visualizations to better fit their screens. Hover tooltips can be automatically added to regions to assist researchers in navigating the visualization. When a circular and linear plot are linked together on the same page, a shaded slice on the circular plot can also be shown to assist researchers in navigating what region on the genome they're zoomed to on the linear plot. As well, double click events are captured to recentre the focused region.

### 2.4 Integration

GenomeD3Plot works best when integrated as a component of a larger platform; it has recently been integrated as the visualization component of a number of analysis pipelines and databases including a new version of IslandViewer (Dhillon *et al.*, 2015) and the Pseudomonas Genome Database (Winsor *et al.*, 2011). As well it’s being used as a visualization tool in the Integrated Infectious Disease Analysis (IRIDA) bacterial genome analysis platform (www.IRIDA.ca).[Fig btv376-F1]

**Fig. 1. btv376-F1:**
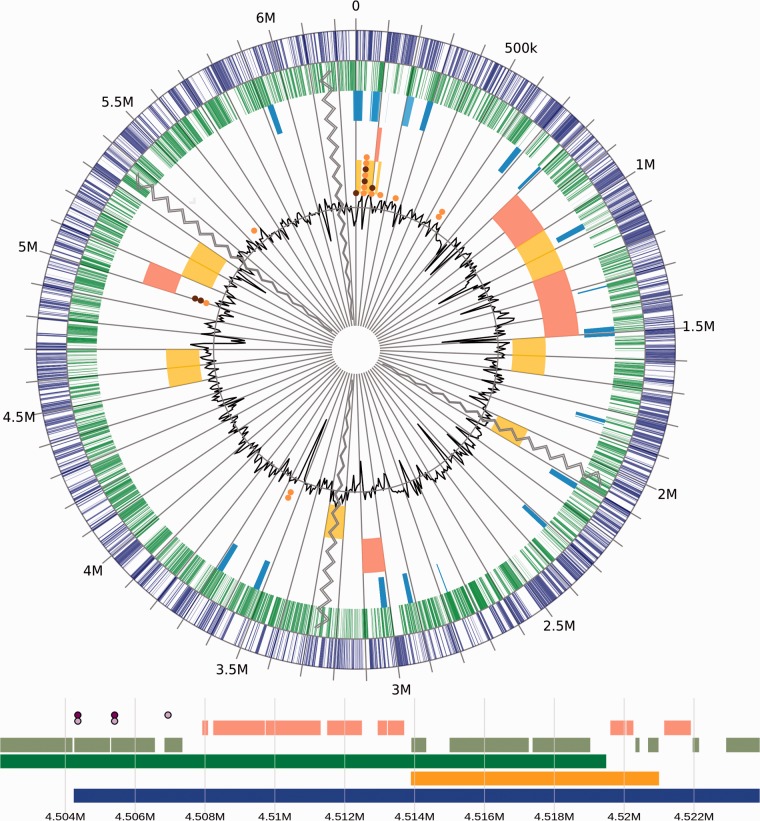
GenomeD3Plot circular and linear visualization of an example genome with annotation data. See www.pathogenomics.sfu.ca/islandviewer/demo and http://bl.ocks.org/lairdm/6a770c94c6793eee660d to see GenomeD3Plot’s more dynamic nature
